# TiRobot-assisted cannulated screw fixation for calcaneal fractures: a meta-analysis of surgical trauma, anatomical restoration, and functional recovery

**DOI:** 10.3389/fsurg.2026.1906865

**Published:** 2026-08-20

**Authors:** Hong Liu, Ruoyan Wang, Guohang Shen, Yinhai Jiang, Li Huang, Junlei Liao, Jie Zhao, Yue Zhou, Yupei Dai, Chuan You

**Affiliations:** 1 Department of Orthopedics, Affiliated Hospital of North Sichuan Medical College, Nanchong, Sichuan, China; 2 North Sichuan Medical College, Nanchong, Sichuan, China; 3 The First Clinical Medical College, Ningxia Medical University, Yinchuan, Ningxia Hui Autonomous Region, China; 4 Department of Anesthesiology, Nanchong Hospital of Beijing Anzhen Hospital Affiliated to Capital Medical University (Nanchong Central Hospital), Nanchong, Sichuan, China; 5 The First Clinical Medical College, Jinan University, Guangzhou, Guangdong, China; 6 Department of Hepatobiliary Surgery, The Affiliated Hospital of North Sichuan Medical College, Nanchong, Sichuan, China

**Keywords:** calcaneal fracture, Cannulated screw, Meta-analysis, minimally invasive surgery, TiRobot

## Abstract

**Background:**

TiRobot-assisted cannulated screw fixation may improve surgical precision and clinical outcomes in calcaneal fractures. This meta-analysis evaluated its effects on surgical trauma, anatomical restoration, functional recovery, and complications compared with conventional fixation.

**Methods:**

PubMed, Embase, Web of Science, Cochrane Library, and CNKI were searched from inception to April 2026. Comparative studies evaluating TiRobot-assisted versus conventional fixation for calcaneal fractures were included. Pooled mean differences (MDs) or odds ratios (ORs) with 95% confidence intervals (CIs) were calculated.

**Results:**

Nine studies involving 509 patients were included. Operative time was comparable between groups (MD = −0.75 min). TiRobot-assisted fixation reduced intraoperative blood loss (MD = −28.22 mL) and improved calcaneal anatomical restoration, including calcaneal height (MD = 1.88 mm), Böhler angle (MD = 1.50°), and Gissane angle (MD = 5.00°). Patients treated with TiRobot assistance showed lower postoperative VAS scores (MD = −0.84 points), higher AOFAS ankle–hindfoot scores (MD = 4.21 points), and fewer overall complications (OR = 0.17).

**Conclusions:**

TiRobot-assisted cannulated screw fixation provides measurable improvements in surgical trauma, anatomical reconstruction, early pain relief, functional recovery, and postoperative safety compared with conventional fixation. Further multicenter randomized controlled trials with long-term follow-up are required to confirm these findings.

## 1 Introduction

Calcaneal fractures are the most common type of tarsal injuries and represent a major category of foot fractures. These injuries are typically caused by high-energy trauma, most commonly resulting from falls from height or motor vehicle accidents (1–3). Inadequate reduction or unstable fixation may lead to post-traumatic subtalar arthritis, malunion of the calcaneus, chronic pain, and gait dysfunction, significantly impairing foot function and patient quality of life (4, 5). Surgical intervention remains the cornerstone for displaced calcaneal fractures. Cannulated screw fixation has emerged as a widely adopted approach due to its minimally invasive nature, reliable stabilization, and facilitation of early rehabilitation (6, 7).

However, the traditional freehand insertion of cannulated screws is largely dependent on the surgeon's clinical expertise and understanding of anatomical landmarks. Repeated intraoperative fluoroscopy is often required to confirm screw positioning, increasing radiation exposure for both patients and clinicians and introducing risks such as screw misplacement, joint penetration, and insufficient fixation (8, 9). Compared with robotic-assisted techniques, conventional open or manually guided minimally invasive procedures typically involve greater surgical exposure, which increases the likelihood of soft tissue complications such as infection, skin necrosis, and hematoma. Moreover, accurate restoration of calcaneal morphology remains difficult with these approaches, as key structural parameters are often suboptimally corrected, potentially compromising postoperative recovery and long-term functional outcomes (10, 11). Therefore, achieving precision, minimally invasive implementation, and safety in cannulated screw fixation for calcaneal fractures represents a critical challenge in contemporary orthopedic trauma surgery.

In recent years, orthopedic robotic navigation systems have been increasingly integrated into minimally invasive fracture surgery by improving spatial localization and trajectory guidance. TiRobot (TINAVI Medical Technologies Co., Ltd., Beijing, China) is a computer-assisted orthopedic navigation platform consisting of a six-degree-of-freedom robotic arm, an optical tracking navigation system, and a patient-specific surgical guide system. The surgical workflow generally involves preoperative three-dimensional reconstruction and trajectory planning based on computed tomography images, followed by intraoperative registration and robotic alignment of the guide according to the planned screw trajectory. During calcaneal fracture fixation, TiRobot does not independently perform screw insertion; rather, it assists surgeons by maintaining accurate positioning and orientation of the guide, thereby facilitating precise percutaneous screw placement (12). The robotic arm provides multiple degrees of freedom for spatial adjustment, allowing flexible positioning within the operative field. During trajectory alignment, certain rotational movements around the guide axis may not directly affect the planned screw pathway, resulting in potential task-level redundancy. This redundancy may facilitate optimal robotic configuration and reduce mechanical interference during surgery. However, the clinical value of this feature mainly lies in improving operational flexibility and consistency rather than providing autonomous surgical capability (13, 14). Current clinical exploration of TiRobot-assisted cannulated screw fixation for calcaneal fractures has shown promising preliminary outcomes. However, most studies are single-center and small-scale, resulting in variable conclusions and lacking high-level evidence. A meta-analysis was performed to quantitatively synthesize data on surgical trauma, fracture reduction quality, postoperative recovery, and complication rates, providing robust evidence to guide the selection of optimal minimally invasive treatment. strategies for calcaneal fractures.

## 2 Materials and methods

### 2.1 Study registration

This systematic review and meta-analysis was registered on the International Prospective Register of Systematic Reviews (PROSPERO), registration number CRD420261374367 (15).

### 2.2 Inclusion and exclusion criteria

#### 2.2.1 Inclusion criteria

- Study Type: Published clinical controlled studies, including randomized controlled trials (RCTs) and non-randomized concurrent or retrospective cohort studies, in English or Chinese.
- Participants: Patients diagnosed with calcaneal fractures by x-ray or CT who were planned for cannulated screw fixation, without restrictions on age, sex, fracture type, or injury mechanism.
- Interventions: The experimental group underwent TiRobot-assisted cannulated screw placement, while the control group received conventional manual or minimally invasive screw fixation.
- Outcomes: operative time, intraoperative blood loss, incision length, length of hospital stay, fluoroscopy frequency, postoperative calcaneal length/width/height, Böhler angle, Gissane angle, fracture healing time, VAS pain score, AOFAS hindfoot function score, overall complications, infection, or skin necrosis.
- Data Availability: Data could be directly extracted or calculated, including mean, standard deviation, sample size, effect size, and 95% confidence interval (CI).

#### 2.2.2 Exclusion criteria

Excluded studies included reviews, systematic reviews, case reports, animal studies, basic science research, and conference abstracts; duplicated publications, studies with data errors, or studies without extractable statistics; studies not using TiRobot or not employing cannulated screw fixation for calcaneal fractures; studies with severe polytrauma, metabolic bone disease, malignancy, or major organ dysfunction affecting outcome assessment; and studies with major design flaws that precluded quality assessment.

### 2.3 Literature search strategy

A comprehensive literature search was conducted in PubMed, Embase, Web of Science, the Cochrane Library, and CNKI from inception to April 2026. The search strategy was developed to maximize retrieval sensitivity by combining database-specific indexing terms with free-text keywords related to robotic-assisted surgery and calcaneal fractures.

The search terms were organized into two conceptual domains: (1) robotic technology-related terms, including “Robot”, “Robots”, “Robotic”, “Robotics”, and “Robot-Assisted”; and (2) disease-related terms, including “calcaneal fracture” and “calcaneal fractures”. These terms were combined using Boolean operators (OR within each domain and AND between domains).

Because TiRobot is a specific orthopedic robotic navigation platform without an independent indexing term in major biomedical databases, relevant free-text terms were additionally applied to ensure comprehensive identification of eligible studies. The detailed search strategies for each database are provided in the Supplementary File.

### 2.4 Study selection and data extraction

Study selection and data extraction were independently conducted by two reviewers. Disagreements were resolved through discussion or consultation with a third reviewer. Extracted information included study characteristics, sample size, interventions, and outcome data.

### 2.5 Quality assessment of included studies

RCTs were evaluated using the Cochrane Risk of Bias tool (16), while non-randomized studies were assessed with the Newcastle–Ottawa Scale and ROBINS-I tool (17).

### 2.6 Statistical analysis

Data synthesis was performed using RevMan 5.4. Continuous variables were expressed as mean differences (MD), and dichotomous outcomes as odds ratios (OR), both with 95% confidence intervals (CI). Heterogeneity was assessed by the *χ*^2^ test and *I*^2^ statistic; a fixed-effects model was applied when *I*^2^ < 50%, otherwise a random-effects model was used.

## 3 Results

### 3.1 Study selection and characteristics of included studies

**Figure 1:**
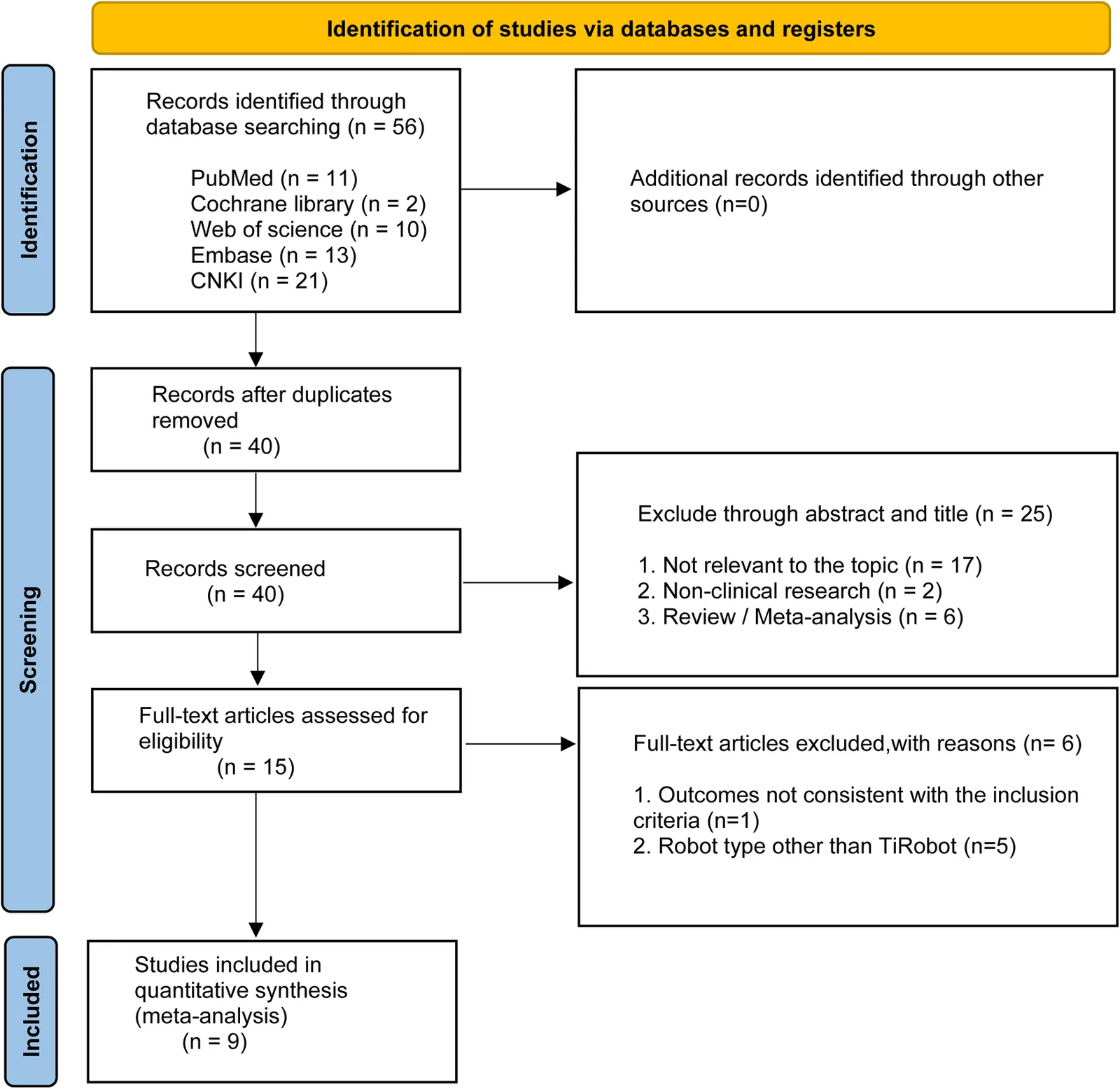
Flow diagram of study selection for the systematic review and meta-analysis.

The detailed selection process is illustrated in Figure 1, and the baseline characteristics of the included studies are summarized in Table 1. These studies involved 509 patients, including 261 in the TiRobot-assisted group and 248 in the conventional treatment group (18–26). A total of 16 outcome measures were extracted, covering perioperative indicators, radiographic parameters, postoperative recovery, and complication-related outcomes (Table 2).

**Table 1:** Baseline patient characteristics and study design summary of the included comparative studies.

Indicator		Yonghong Dai	Jing Wang	Huan Guo	Jinfeng Zhang	Xiaorui Zhou	Liang Li	Tianyi Zou	Xinwei Yuan	Junhua Xu
Publication year		2025	2023	2025	2023	2026	2025	2023	2021	2024
Cases (n)	RA	10	15	54	18	50	49	38	19	8
	CF	10	13	44	14	50	45	35	25	12
Gender (n) (female/male)										
	RA	2/8	3/12	16/38	4/14	22/28	8/41		6/13	1/7
	CF	2/8	1/12	12/32	4/10	21/29	6/39		7/18	3/9
Age (years) (mean ± SD)										
	RA	49.80 ± 17.26	45.20 ± 11.40	47.20 ± 11.87	44.6 ± 9.6	34.67 ± 3.52	38.33 ± 9.67	45(mean)	50.2(mean)	48.75 ± 9.49
	CF	47.10 ± 15.06	46.15 ± 9.25	46.08 ± 10.70	46.5 ± 9.4	34.11 ± 3.49	38.45 ± 9.81	45(mean)	53.5(mean)	50.25 ± 11.15
BMI, kg/m^2^ (mean ± SD)										
	RA	24.73 ± 3.18			23.4 ± 3.5					
	CF	22.45 ± 3.38			25.3 ± 6.0					
Side of injured (left/right)										
	RA	3/7		30/24	10/8	26/24	23/26		11/8	3/5
	CF	6/4		23/21	6/8	28/22	21/24		14/11	4/8
Sanders classification (Type II/Type III/Type IV)										
	RA	3/7/0		28/26/0		27/23/0	25/24/0		12/7/0	6/2/0
	CF	5/5/0		25/19/0		26/24/0	23/22/0		18/7/0	9/3/0
Follow-up time (months) (mean ± SD)										
	RA	28.47 ± 6.02	3	12	3	12	12	6	9.5 (mean)	3–6
	CF	28.43 ± 5.81	3	12	3	12	12	6	9.5 (mean)	3–6
Study design		RCS	RCS	RCS	RCS	RCT	RCS	RCT	RCS	RCS

RA, robot-assisted; CF, conventional freehand; *N*, sample size.

The names listed are all authors of the included publications, not patients or research participants.

**Table 2:** Pooled perioperative surgical parameters and postoperative clinical outcomes from nine comparative studies.

Author		Operative time (minutes)	Intraoperative blood loss (mL)	Incision length (cm)	Length of hospital stay (days)	Intraoperative fluoroscopy frequency	Calcaneal length (mm)	Calcaneal width (mm)	Calcaneal height (mm)	Böhler angle	Gissane angle
Yonghong Dai	RA	113.8 ± 37.36	14.62 ± 19.35	2.37 ± 0.86	7.83 ± 3.66		76.52 ± 4.97	36.75 ± 5.8	47.24 ± 4.47	30.84 ± 9.65	125.48 ± 4.31
	CF	73.5 ± 16.21	47.17 ± 77.4	7.63 ± 0.86	11.2 ± 4.95		75.76 ± 3.26	37.57 ± 2.26	48.06 ± 4.17	24.43 ± 6.38	117.22 ± 11.67
Jing Wang	RA				2.87 ± 1.13			40.63 ± 2.31		27.16 ± 3.45	
	CF				5.46 ± 2.82			39.54 ± 2.6		26.16 ± 3.96	
Huan Guo	RA	50.53 ± 6.46	6.53 ± 2.16		5.72 ± 1.04		83.59 ± 4.06	41.05 ± 4.35	41.53 ± 3.72	28.59 ± 3.07	132.88 ± 5.38
	CF	56.72 ± 10.12	14.27 ± 4.19		6.31 ± 1.66		81.57 ± 5.7	43.15 ± 4.88	39.79 ± 4.05	27.22 ± 3.73	130.61 ± 5.42
Jinfeng Zhang	RA		4.64 ± 2.41		4.64 ± 2.41	4.22 ± 2.41	82.83 ± 4.83	39.6 ± 3.06	46.08 ± 4.67	26.35 ± 5.07	
	CF		15.56 ± 6.43		6 ± 3.3	8.38 ± 1.89	81.43 ± 8.07	40.14 ± 6.26	44.1 ± 2.97	27.42 ± 3.46	
Xiaorui Zhou	RA	85.79 ± 8.65	21.05 ± 2.23		5.62 ± 0.63	36.12 ± 3.74	77.45 ± 7.89		40.25 ± 4.18	26.38 ± 2.72	131.26 ± 13.51
	CF	65.41 ± 6.72	46.11 ± 4.72		8.79 ± 0.94	38.18 ± 4.37	73.26 ± 7.61		37.77 ± 3.79	24.89 ± 2.57	123.88 ± 13.37
Liang Li	RA	70.68 ± 8.04	23.48 ± 3.92					34.61 ± 2.25		31.45 ± 1.79	135.14 ± 6.76
	CF	72.97 ± 18.68	71.55 ± 9.28					35.13 ± 2.46		31.22 ± 2.4	134.84 ± 6.51
Tianyi Zou	RA		10.27 ± 7.13		5 ± 1.54			24.44 ± 1.03		32.41 ± 4.57	130.24 ± 4.7
	CF		51.07 ± 20.87		11.68 ± 3.79			25.27 ± 1.12		31.75 ± 5.43	128.2 ± 4.85
Xinwei Yuan	RA	78.31 ± 13.05				3.05 ± 0.22				35.36 ± 4.04	
	CF	59.92 ± 6.53				9.92 ± 2.05				36.12 ± 4.08	
Junhua Xu	RA	80.13 ± 3.271	14.38 ± 7.289	3.25 ± 0.463	9.5 ± 3.423	4.25 ± 1.282					
	CF	69.25 ± 3.729	51.67 ± 19.46	7.667 ± 1.435	14.83 ± 3.04	6.667 ± 1.614					

Author		Fracture Healing Time (weeks)	VAS Pain Score	AOFAS Ankle-Hindfoot Score	Overall Complication Rate	Infection	Skin Necrosis
Yonghong Dai	RA	12 ± 6.88	0.37 ± 0.86	95.28 ± 9.46	2	0	
	CF	17.68 ± 4.2	1.72 ± 2.8	85.24 ± 10.32	8	2	
Jing Wang	RA			77.13 ± 4.78	4	3	
	CF			75.15 ± 4.78	4	3	
Huan Guo	RA	13.7 ± 1.21	2.87 ± 0.69	78.23 ± 5.34	1	0	
	CF	14.76 ± 1.58	3.87 ± 0.72	75.72 ± 6.8	6	0	
Jinfeng Zhang	RA			84.2 ± 5.5	0	0	0
	CF			79.2 ± 6.2	8	3	5
Xiaorui Zhou	RA	12.08 ± 1.32	2.85 ± 0.31		5		
	CF	13.48 ± 1.44	3.47 ± 0.38		7		
Liang Li	RA			77.13 ± 4.78	0	0	
	CF			75.15 ± 4.78	16	12	
Tianyi Zou	RA		2 ± 3.08	79.92 ± 9.58	2		0
	CF		3.36 ± 2.32	72 ± 6.9	13		3
Xinwei Yuan	RA	15.63 ± 2.36		90 ± 4.5	0		
	CF	17.08 ± 2.62		85.7 ± 4.5	2		
Junhua Xu	RA	15.13 ± 1.727		77.13 ± 3.441	0		
	CF	16.33 ± 1.723		70.83 ± 3.512	0		

### 3.2 Quality assessment of included studies

**Figure 2:**
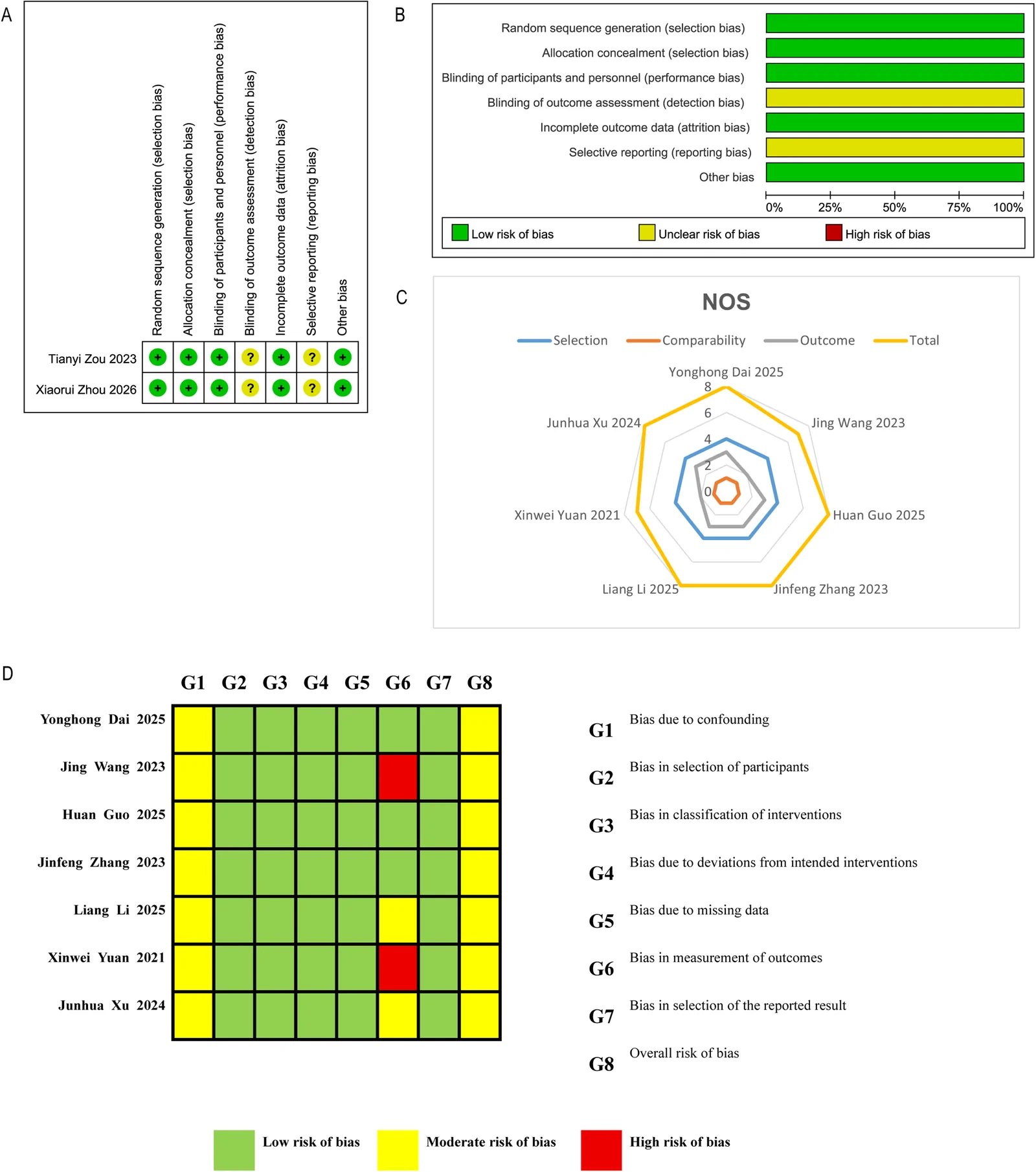
Methodological quality and risk-of-bias assessment of the included studies. **(A)** Risk-of-bias graph for RCTs; **(B)** risk-of-bias summary for RCTs; **(C)** quality assessment of retrospective cohort studies based on the NOS; **(D)** overall risk-of-bias evaluation of non-randomized studies using the ROBINS-I tool.

The methodological quality of the included studies was rated as moderate to high overall. Detailed results are shown in Figure 2.

### 3.3 Perioperative outcomes

#### 3.3.1 Operative time

**Figure 3:**
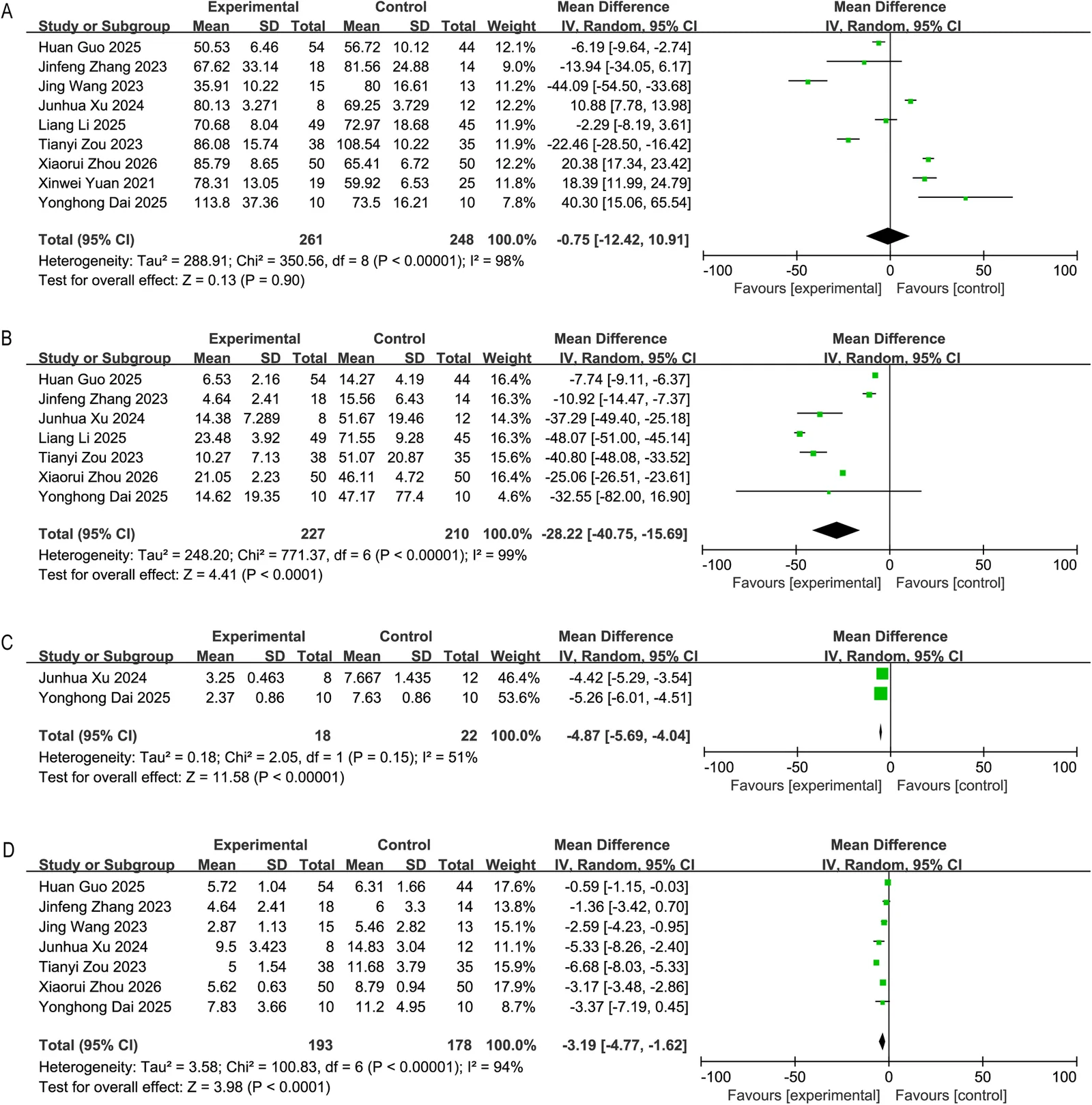
Meta-analysis of perioperative outcomes. **(A)** Operative time; **(B)** intraoperative blood loss; **(C)** incision length; **(D)** length of hospital stay.

Operative duration was available from nine reports, covering 261 individuals in the TiRobot arm and 248 in the conventional arm. When synthesized, the evidence did not indicate a meaningful between-technique difference in operative time (MD = −0.75, 95% CI: −12.42 to 10.91, *P* > 0.05) (Figure 3A).

#### 3.3.2 Intraoperative blood loss

Seven reports contributed data on intraoperative blood loss, representing 227 individuals treated with TiRobot assistance and 210 treated conventionally. The pooled estimate favored robotic assistance, with a clear reduction in blood loss (MD = −28.22, 95% CI: −40.75 to −15.69, *P* < 0.05) (Figure 3B).

#### 3.3.3 Incision length

Incision length was documented in two comparisons, involving 18 individuals in the TiRobot cohort and 22 in the conventional cohort. The robotic technique was associated with a markedly shorter incision (MD = −4.87, 95% CI: −5.69 to −4.04, *P* < 0.05) (Figure 3C).

#### 3.3.4 Length of hospital stay

Length of hospitalization was reported in seven studies, including 193 individuals in the TiRobot cohort and 178 in the conventional cohort. Combined analysis showed that robotic assistance was linked to a shorter hospital stay (MD = −3.19, 95% CI: −4.77 to −1.62, *P* < 0.05) (Figure 3D).

#### 3.3.5 Intraoperative fluoroscopy frequency

**Figure 4:**
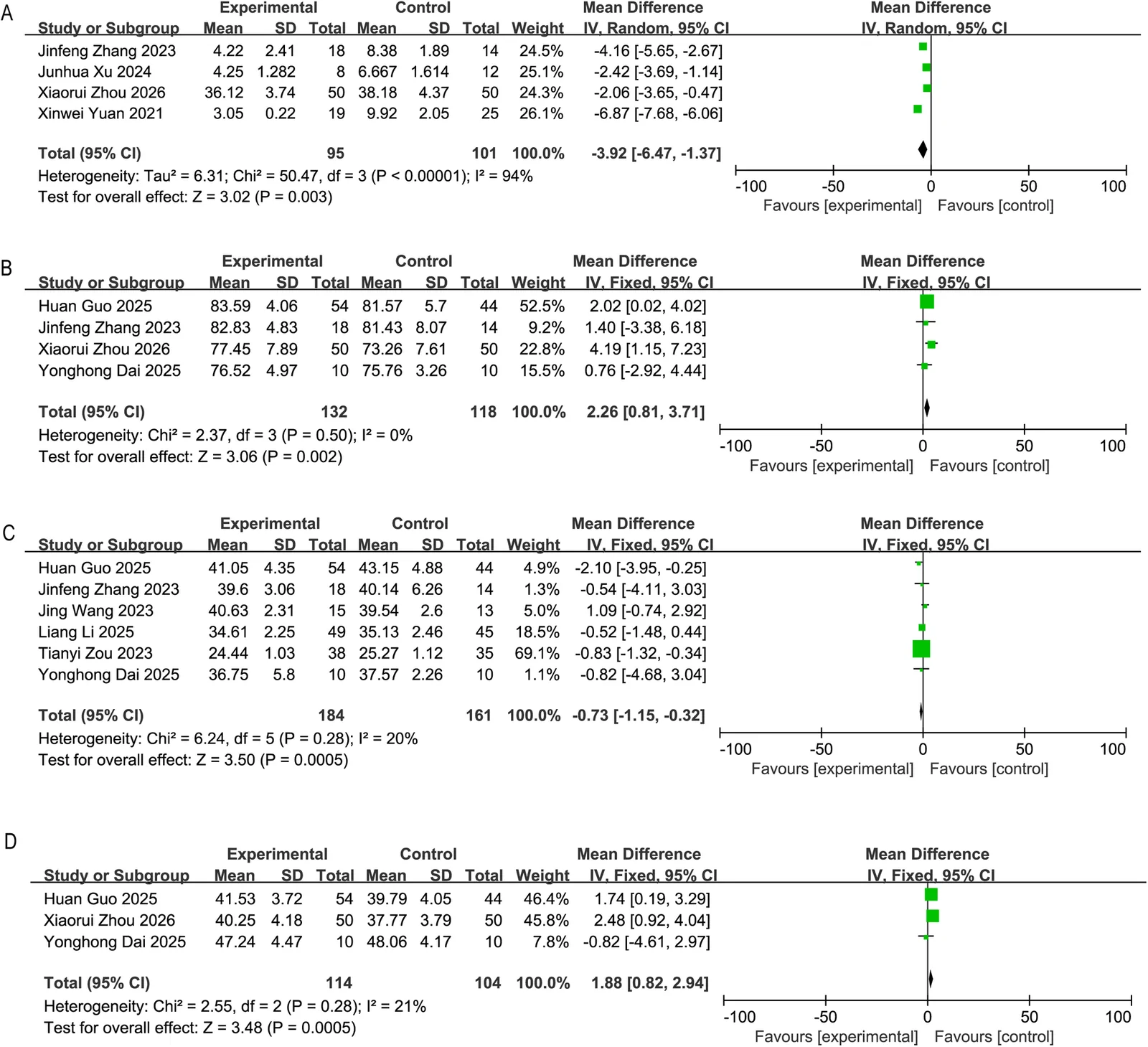
Meta-analysis of intraoperative and postoperative radiological parameters. **(A)** Intraoperative fluoroscopy frequency; **(B)** postoperative calcaneal length; **(C)** postoperative calcaneal width; **(D)** postoperative calcaneal height.

Four studies quantified intraoperative fluoroscopy use, with 95 individuals in the TiRobot arm and 101 in the conventional arm. The robotic strategy required fewer fluoroscopy exposures overall (MD = −3.92, 95% CI: −6.47 to −1.37, *P* < 0.05) (Figure 4A).

### 3.4 Postoperative calcaneal morphometric parameters

#### 3.4.1 Postoperative calcaneal length

Postoperative calcaneal length was examined in four studies that included 132 individuals receiving TiRobot-assisted fixation and 118 receiving conventional fixation. The summary effect showed better restoration of calcaneal length with the robotic method (MD = 2.26 mm, 95% CI: 0.81–3.71, *P* < 0.05) (Figure 4B).

#### 3.4.2 Postoperative calcaneal width

Six studies evaluated postoperative calcaneal width, encompassing 184 individuals in the TiRobot cohort and 161 in the conventional cohort. After pooling, the robotic approach produced a significantly narrower postoperative width, indicating superior correction of lateral expansion (MD = −0.73 mm, 95% CI: −1.15–-0.32, *P* < 0.05) (Figure 4C).

### 3.5 Core radiographic parameters reflecting anatomical restoration

**Figure 5:**
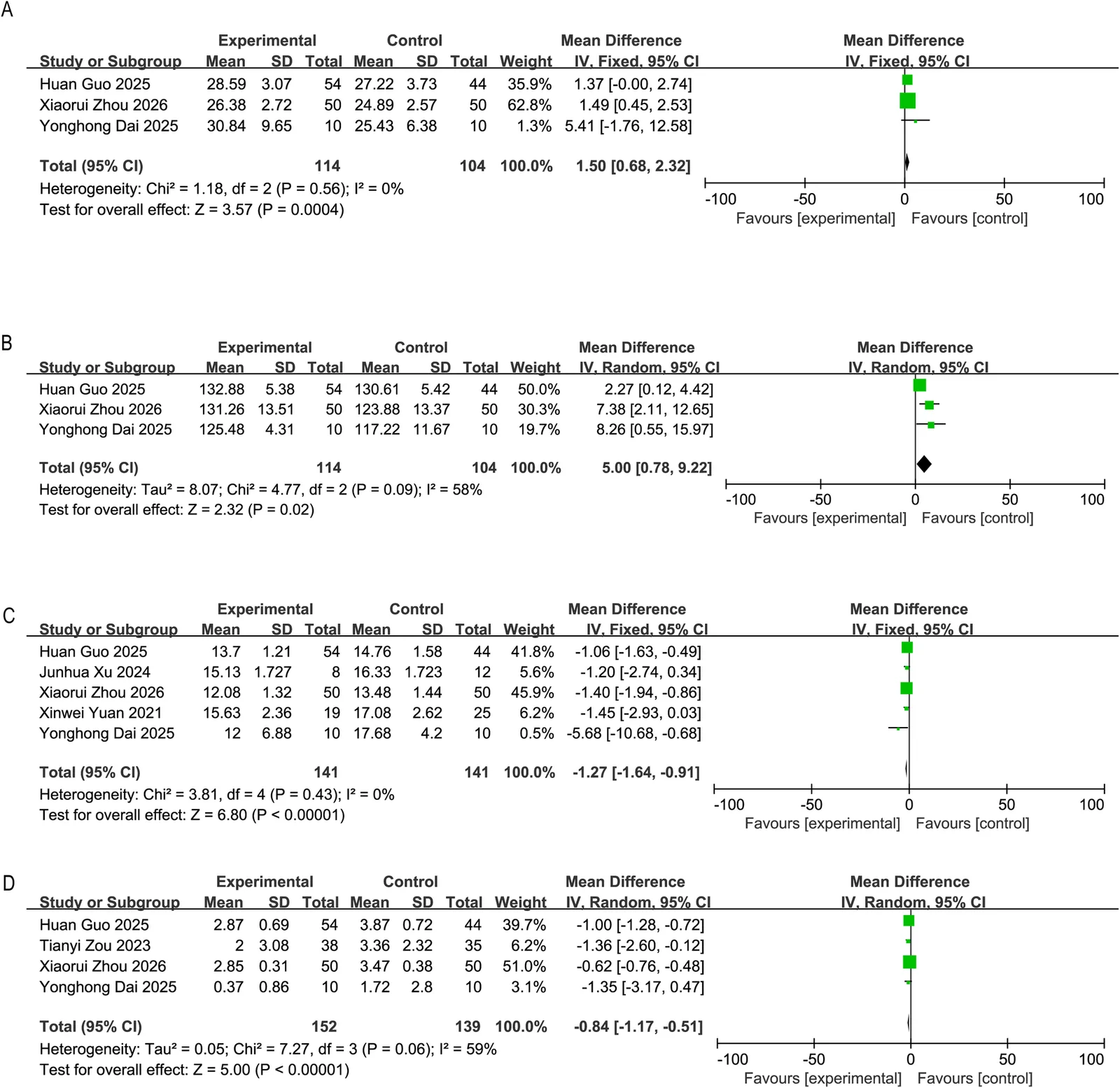
Meta-analysis of postoperative radiographic and clinical recovery outcomes. **(A)** Postoperative Böhler angle; **(B)** postoperative Gissane angle; **(C)** fracture healing time; **(D)** postoperative VAS pain score.

To provide a consistent evaluation of three-dimensional anatomical reconstruction, studies that simultaneously reported the three key radiographic parameters, including postoperative calcaneal height, Böhler angle, and Gissane angle, were selected for integrated analysis. A total of three studies involving 218 patients (114 patients in the TiRobot-assisted group and 104 patients in the conventional group) were included. Regarding postoperative calcaneal height restoration, TiRobot-assisted fixation demonstrated significantly improved recovery compared with conventional fixation (MD = 1.88 mm, 95% CI: 0.82–2.94, *P* < 0.05) (Figure 4D), indicating superior restoration of vertical calcaneal morphology. For Böhler angle correction, patients receiving TiRobot-assisted fixation achieved a significantly greater postoperative angle compared with the conventional group (MD = 1.50°, 95% CI: 0.68–2.32, *P* < 0.05) (Figure 5A), suggesting enhanced restoration of posterior facet alignment and calcaneal height. Similarly, TiRobot-assisted fixation resulted in improved recovery of the Gissane angle (MD = 5.00°, 95% CI: 0.78–9.22, *P* < 0.05) (Figure 5B), reflecting better reconstruction of the anterior process and overall calcaneal anatomical configuration. Collectively, these findings indicate that TiRobot-assisted fixation provides more accurate three-dimensional restoration of calcaneal morphology compared with conventional techniques.

### 3.6 Postoperative recovery and functional outcomes

#### 3.6.1 Fracture healing time

Fracture-healing time was described in five studies, each treatment arm contributing 141 individuals. The pooled estimate indicated faster healing after TiRobot-assisted fixation (MD = −1.27, 95% CI: −1.64 to −0.91, *P* < 0.01) (Figure 5C). This pattern is consistent with the possibility that a less disruptive procedure and more stable fixation create a more favorable environment for repair.

#### 3.6.2 Postoperative Vas pain score

Postoperative pain, assessed with the visual analog scale (VAS), was reported in four studies involving 152 individuals in the TiRobot arm and 139 in the conventional arm. Pain scores were significantly lower after robotic assistance (MD = −0.84, 95% CI: −1.17 to −0.51, *P* < 0.01) (Figure 5D), supporting a more comfortable early recovery course.

#### 3.6.3 Postoperative AOFAS ankle–hindfoot score

**Figure 6:**
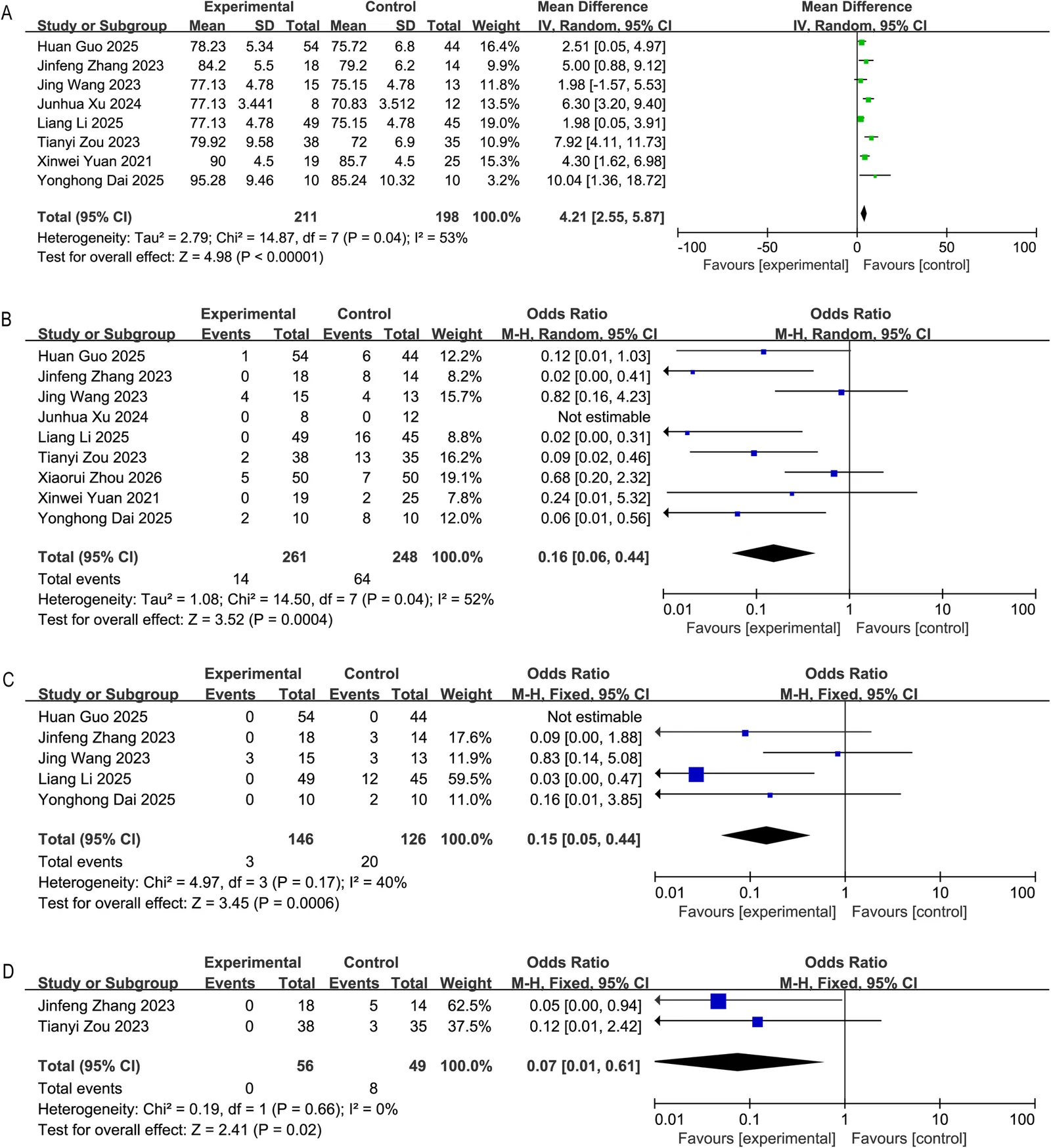
Meta-analysis of functional outcomes and postoperative complications. **(A)** Postoperative AOFAS ankle-hindfoot score; **(B)** overall complication rate; **(C)** infection-related complications; **(D)** incidence of skin necrosis.

Eight studies used the AOFAS ankle–hindfoot scale to evaluate functional recovery, covering 211 individuals in the TiRobot cohort and 198 in the conventional cohort. Functional outcomes favored the robotic-assisted approach, with a significantly higher postoperative AOFAS ankle–hindfoot score compared with conventional fixation (MD = 4.21, 95% CI: 2.55 to 5.87, *P* < 0.01) (Figure 6A). This difference represents a quantifiable improvement in postoperative functional recovery associated with TiRobot-assisted fixation.

### 3.7 Postoperative complications

#### 3.7.1 Overall complication rate

Overall postoperative complications were analyzed in eight studies, representing 251 individuals managed with TiRobot assistance and 238 managed conventionally. The summary odds ratio showed a lower complication burden in the robotic cohort (OR = 0.17, 95% CI: 0.06–0.54, *P* < 0.05) (Figure 6B), indicating a meaningful safety advantage.

#### 3.7.2 Infection-related complications

Five studies contributed data on infection-related events, including 146 individuals in the TiRobot arm and 126 in the conventional arm. The risk of infection was substantially lower with robotic assistance (OR = 0.15, 95% CI: 0.05–0.44, *P* < 0.01) (Figure 6C).

#### 3.7.3 Skin necrosis-related complications

Skin necrosis was reported in two studies, with 56 individuals in the TiRobot cohort and 49 in the conventional cohort. The combined estimate strongly favored the robotic technique, showing a lower incidence of skin necrosis (OR = 0.07, 95% CI: 0.01–0.61, *P* < 0.05) (Figure 6D), which is in line with improved soft-tissue preservation.

## 4 Discussion

This meta-analysis indicates that TiRobot-assisted cannulated screw fixation provides clear advantages over conventional techniques in several key clinical aspects. The robotic approach was associated with reduced intraoperative blood loss, smaller incisions, shorter hospital stay, and fewer fluoroscopy exposures, suggesting decreased surgical trauma and radiation exposure. In terms of anatomical restoration, improved outcomes were observed in calcaneal length, height, Böhler angle, and Gissane angle, along with better correction of calcaneal width, reflecting higher reduction accuracy. Additionally, patients in the TiRobot group showed faster fracture healing, lower postoperative pain, and higher AOFAS scores, indicating enhanced recovery. The overall complication rate, including infection and skin necrosis, was also significantly lower. Collectively, these findings support the superiority of TiRobot-assisted fixation in improving surgical precision, recovery outcomes, and safety in the treatment of calcaneal fractures.

Calcaneal fractures are the most common tarsal injuries and are usually caused by high-energy trauma, frequently involving the subtalar joint and complex structural damage. Inadequate reduction or unstable fixation can result in complications such as post-traumatic arthritis, malunion, chronic pain, and gait impairment, ultimately affecting long-term function and quality of life (27, 28). Cannulated screw fixation is widely used due to its minimally invasive nature and reliable stability. However, conventional freehand techniques rely heavily on surgical experience and repeated fluoroscopy, increasing the risk of screw misplacement, soft tissue injury, and radiation exposure. Moreover, precise anatomical reduction remains difficult to achieve with traditional methods (6, 10). With the rapid advancement of orthopedic robotic technologies, the integration of precision navigation and minimally invasive surgery has provided new opportunities for improving surgical outcomes. The TiRobot system enables preoperative three-dimensional planning and intraoperative real-time navigation, allowing for individualized trajectory design and highly accurate screw placement. This approach not only enhances fixation precision but also minimizes soft tissue disruption and reduces reliance on repeated fluoroscopic guidance. The present study, by synthesizing current comparative clinical evidence, further substantiates the clinical value of robotic-assisted techniques in calcaneal fracture treatment and provides important evidence to support the broader adoption of precision and minimally invasive strategies in orthopedic trauma surgery.

Perioperative parameters serve as critical indicators for evaluating surgical invasiveness and procedural efficiency. In the present study, no statistically significant difference in operative time was observed between the two groups, a finding that contrasts with conventional expectations. This outcome can be explained by the procedural characteristics of robotic-assisted surgery. Although the TiRobot system requires additional preoperative steps, including image acquisition, data registration, and trajectory planning, intraoperative navigation enables precise screw placement in a single attempt. This reduces the need for repeated puncture and fluoroscopic adjustments commonly encountered in conventional procedures. Consequently, the time invested in preoperative planning is effectively offset by enhanced intraoperative efficiency, resulting in a comparable overall operative duration. These findings suggest that robotic assistance optimizes surgical workflow without compromising time efficiency. Notably, the TiRobot-assisted approach demonstrated significant advantages in intraoperative blood loss, incision length, length of hospital stay, and fluoroscopy frequency. These improvements are largely attributable to its minimally invasive nature. By leveraging precise navigation and robotic arm guidance, screw insertion can be accomplished through smaller incisions without extensive soft tissue and periosteal dissection. This approach preserves local vascular supply and maintains soft tissue integrity at the fracture site.

Accurate restoration of calcaneal anatomy is fundamental to re-establishing normal foot biomechanics and achieving favorable long-term outcomes. Previous studies have demonstrated that alterations in calcaneal morphology—such as decreased length, increased width, reduced height, and abnormal Böhler and Gissane angles—can disrupt subtalar joint congruency and load distribution, ultimately predisposing patients to post-traumatic arthritis and chronic pain (29, 30). The present analysis confirms that TiRobot-assisted fixation provides superior restoration of these key anatomical parameters, with a more normalized calcaneal width, highlighting its advantage in achieving precise three-dimensional reduction. From a mechanistic perspective, conventional freehand screw placement relies heavily on two-dimensional fluoroscopic imaging and surgeon experience, which may not adequately capture the complex three-dimensional geometry of the calcaneus. Intraoperative real-time navigation further ensures accurate execution of the planned procedure. This transition from an experience-dependent approach to a data-driven and image-guided strategy enhances the reproducibility and precision of fracture reduction, thereby facilitating restoration of articular congruity and biomechanical stability.

Postoperative recovery and functional restoration represent the ultimate endpoints for evaluating the clinical effectiveness of surgical interventions. In the present study, the TiRobot-assisted group demonstrated significantly superior outcomes in fracture healing time, postoperative pain (VAS score), and AOFAS ankle–hindfoot functional scores compared with the conventional group. These improvements are likely attributable to the combined effects of reduced surgical invasiveness, enhanced reduction accuracy, and improved fixation stability. From a biological perspective, the minimally invasive nature of robotic-assisted surgery minimizes periosteal disruption and soft tissue damage, thereby preserving the local vascular supply at the fracture site. This optimized biological environment is conducive to bone regeneration and may account for the observed acceleration in fracture healing. From a biomechanical standpoint, accurate anatomical reduction coupled with stable internal fixation reduces micromotion at the fracture interface, thereby mitigating nociceptive stimulation associated with mechanical instability. In addition, the smaller surgical incision contributes to reduced postoperative wound pain, collectively resulting in lower VAS scores. Importantly, the combination of precise anatomical restoration, stable fixation, and a lower complication profile enables earlier initiation of functional rehabilitation. This early mobilization helps prevent complications associated with prolonged immobilization, such as joint stiffness, muscle atrophy, and functional decline. The synergistic effect of these factors ultimately leads to improved ankle–hindfoot function, as reflected by the significantly higher AOFAS scores. These findings underscore that robotic-assisted techniques not only enhance structural reconstruction but also provide substantial benefits in functional recovery and overall quality of life. Complication rates are a key indicator of surgical safety. In this study, the TiRobot-assisted group showed significantly lower rates of overall complications, infection, and skin necrosis compared with the conventional group, with the greatest reduction observed in skin necrosis, suggesting better soft tissue preservation. Beyond statistical significance, the magnitude of these improvements provides important information regarding clinical interpretation. The pooled increase of 4.21 points in AOFAS score and reduction of 0.84 points in VAS pain score suggest that TiRobot-assisted fixation may provide meaningful early functional benefits. Similarly, improvements in calcaneal height, Böhler angle, and Gissane angle reflect enhanced anatomical reconstruction; however, the direct relationship between individual radiographic changes and long-term patient-reported outcomes requires further validation through prospective studies with extended follow-up.

From a mechanistic perspective, robotic-assisted surgery integrates precise navigation with minimally invasive techniques, enabling screw placement through smaller incisions while minimizing soft tissue disruption. This approach preserves local vascular integrity and reduces wound tension, thereby mitigating the underlying causes of wound healing complications, infection, and skin necrosis. Furthermore, the use of image-guided, high-precision screw placement reduces the risk of implant-related complications, such as intra-articular screw penetration, inadequate fixation, and hardware failure. Collectively, these factors contribute to a substantial improvement in overall surgical safety. Taken together, TiRobot-assisted cannulated screw fixation aligns well with the evolving paradigm of precision, minimally invasive, and safety-oriented orthopedic surgery, and represents a promising treatment option for calcaneal fractures.

## 5 Limitations

Several limitations should be noted. First, the number of included RCTs was limited, with most studies being non-randomized, which may introduce bias and affect internal validity. Second, significant heterogeneity existed in some outcomes, likely due to differences in study design, surgical experience, sample size, rehabilitation protocols, and fracture classification; although a random-effects model was used, its impact cannot be fully eliminated. Finally, follow-up periods were generally short and mainly focused on early outcomes, with insufficient evidence regarding long-term function and post-traumatic arthritis. Therefore, the long-term effectiveness of TiRobot-assisted techniques remains uncertain.

## 6 Conclusion

This study is, to our knowledge, the first meta-analysis to comprehensively evaluate the efficacy and safety of TiRobot-assisted cannulated screw fixation for calcaneal fractures. The findings indicate that operative time is comparable between the two approaches, while the robotic technique shows advantages in perioperative outcomes, anatomical restoration, and postoperative recovery. In addition, complication rates, particularly infection and skin necrosis, were significantly lower, suggesting an improved safety profile. Overall, this method demonstrates better clinical performance than conventional techniques.

## Data Availability

The original contributions presented in the study are included in the article/Supplementary Material, further inquiries can be directed to the corresponding author/s.
