# Supplementary material for: TiRobot-assisted cannulated screw fixation for calcaneal fractures: a meta-analysis of surgical trauma, anatomical restoration, and functional recovery
Source: Front Surg. 2026 Aug 20;13:1906865. doi: 10.3389/fsurg.2026.1906865 (PMC13538863; doi:10.3389/fsurg.2026.1906865)
Supplement: Supplementary file 1 [file Table1.docx]

| **Pubmed** |  |
| --- | --- |
|  | (((((Robot) OR (Robots)) OR (Robotic)) OR (Robotics)) OR (Robot-Assisted)) AND ((calcaneal fractures[Title/Abstract]) OR (calcaneal fracture[Title/Abstract])) |

| **Web of science** |  |
| --- | --- |
| #3 | #2 AND #1 |
| #2 | ((((TS=(Robot)) OR TS=(Robots)) OR TS=(Robotic)) OR TS=(Robotics)) OR TS=(Robot-Assisted) |
| #1 | (TS=(calcaneal fractures)) OR TS=(calcaneal fracture) |

| **Embase** |  |
| --- | --- |
| #3 | #2 AND #1 |
| #2 | 'robot':ab,ti OR 'robots':ab,ti OR 'robotic':ab,ti OR 'robotics':ab,ti OR 'Robot-Assisted':ab,ti |
| #1 | 'calcaneal fractures':ab,ti OR 'calcaneal fracture':ab,ti |

| **Cochrane** |  |
| --- | --- |
| #3 | #2 AND #1 |
| #2 | (Robot):ti,ab,kw OR (Robots):ti,ab,kw OR (Robotic):ti,ab,kw OR (Robotics):ti,ab,kw OR ('Robot-Assisted):ti,ab,kw |
| #1 | (calcaneal fractures):ti,ab,kw OR (calcaneal fracture):ti,ab,kw |

| **CNKI** |  |
| --- | --- |
|  | calcaneal fracture and Robot |
